# Reliability and validity of the *Sidaamu Afoo* version of the pelvic organ prolapse symptom score questionnaire

**DOI:** 10.1186/s12905-023-02478-x

**Published:** 2023-06-20

**Authors:** Melese Siyoum, Wondwosen Teklesilasie, Rahel Nardos, Biniyam Sirak, Ayalew Astatkie

**Affiliations:** 1 Department of Midwifery, College of Medicine and Health Sciences, Hawassa University, Hawassa, Ethiopia; 2 School of Public Health, College of Medicine and Health Sciences, Hawassa University, Hawassa, Ethiopia; 3 Department of Obstetrics and Gynecology, and Women’s Health, University of Minnesota, Minneapolis, USA; 4 Yirgalem Hamlin Fistula Center, Yirgalem, Ethiopia

**Keywords:** Pelvic organ prolapse, POP-SS, Reliability, Validity, Psychometric properties, *Sidaamu Afoo*, Ethiopia

## Abstract

**Background:**

Both for clinical and research purposes, it is critical that clinicians and researchers use a tool that is trans-culturally adapted and tested for its psychometric properties. The English version of the Pelvic Organ Prolapse Symptom Score (POP-SS) questionnaire was developed in 2000. Since then it has been translated into other languages and verified. However, the tool has not been adapted for use in *Sidaamu Afoo* language in the Sidama Region of Ethiopia.

**Objective:**

This study aimed to translate and adapt the Pelvic Organ Prolapse Symptom Score questionnaire into *Sidaamu Afoo* and evaluate its psychometric properties.

**Methods:**

A total of 100 women with symptomatic prolapse completed version-2 of the POP-SS questionnaire during the first round of interviews, and 61 of them completed the questionnaire during the second round of interviews (to establish the test-retest reliability). We adapted the scale translation process recommended by Beaton and his colleagues. The content validity was assessed using the content validity index and the construct validity was done based on exploratory factor analysis using the principal component analysis model. The criterion validity was evaluated by using the Kruskal-Wallis test based on stages of the prolapse established via pelvic examination. The internal consistency reliability of the scale was assessed using Cronbach’s alpha value, and test-retest reliability was evaluated using the intraclass correlation coefficient.

**Results:**

The questionnaire was successfully translated to *Sidaamu Afoo*, and achieved a good content validity index (0.88), high internal consistency (Cronbach’s alpha of 0.79), and test-retest reliability (an intraclass correlation coefficient of 0.83). The exploratory factor analysis revealed two factors based on an eigenvalue of 1. The two factors explained 70.6% of the common variance, and each item loaded well (0.61 to 0.92) to its corresponding factor. There is a significant difference in the median score of prolapse symptoms across different stages of prolapse (Kruskal-Wallis χ^2^, 17.5, p < 0.001).

**Conclusion:**

The *Sidaamu Afoo* version of the POP-SS tool is valid and reliable. Further studies that involve a balanced number of women in each stage of prolapse are needed to avoid the ceiling and floor effects.

**Supplementary Information:**

The online version contains supplementary material available at 10.1186/s12905-023-02478-x.

## Introduction

Pelvic Organ Prolapse (POP) is the descent of one or more of the vaginal walls, cervix, uterus, bladder, or rectums into or out of the vaginal canal [1–4]. It is a complex disorder with both physical and functional elements [5] which require early treatment and has a substantial influence on the quality of life and psychological well-being of women [5–7]. The overall prevalence reaches up to 50% when diagnosed through vaginal examination [8]. The risk is increased in low and middle-income countries due to early marriage, heavy physical work and poor nutrition [9]. A prevalence of 60.9% was reported from Nepal [10]. A recent community-based study from northern Ethiopia showed that the prevalence of POP was 56.3% on pelvic examination and 26.3% based on symptoms [11]. Overall, it is challenging to establish the population-level prevalence of POP as the prevalence varies widely based on the tool used to diagnose the condition and population (symptomatic or asymptomatic) [12]. Therefore clinicians and researchers need to use a standard tool and criteria to accurately diagnose this disorder [13].

Hagen and colleagues developed a Pelvic Organ Prolapse Symptom Score (POP-SS) tool for English-speaking women in 2000 and published its psychometric properties in 2009 and 2010 [14, 15]. However, to use tools in other languages, they should be translated, cross-culturally adapted and their psychometric properties tested [16–18]. This helps the comparison of outcome measures and the accuracy of measurements [19]. So far, the POP-SS tool has been successfully translated into Turkish [20], Amharic [21], and Chinese [22] versions, and has been found to have acceptable psychometric properties. Evaluation of the international use of the questionnaire shows that the tool is commonly used in high-income countries both for clinical and research purposes and is also in use in low and middle-income countries [23]. The tool was used both for assessment of common prolapse symptoms [14] and for outcome measure after intervention [24]. The POP-SS is a condition-specific and brief index of symptoms that measures the extent of prolapse symptoms. However, in the current study area, its use for the assessment of prolapse symptoms by clinicians is inconsistent and not all items are used for prolapse assessment.

To our knowledge, no published study has evaluated the psychometric properties of this tool in *Sidaamu Afoo*, which is the mother tongue of the majority of the population in the Sidama Region of Ethiopia. Therefore, this study was designed to translate and adapt the POP-SS questionnaire into *Sidaamu Afoo* and evaluate its psychometric properties (reliability and validity) among women with POP symptoms.

## Methods and materials

This study involved two phases. Initially, the original tool was translated to the target language, pretested, and data used to assess its psychometric properties other than test-retest reliability were collected. In the second phase, selected participants were interviewed again to evaluate the questionnaire’s test-retest reliability.

### The original POP-SS questionnaire

The original version of the POP-SS has seven questions with a five-point Likert scale which range from zero to four (0 = never felt symptom, 1 = occasionally, 2 = sometimes, 3 = most of the time, and 4 = all of the time), a higher score indicating severe symptoms [14]. The participants are asked how often they have had the following symptoms in the last four weeks: (1) A feeling of something coming down from the vagina? (2) Uncomfortable feeling in the vagina that worsens when standing? (3) A heaviness feeling in the lower abdomen? (4) A dragging feeling in the lower back? (5) A difficulty in emptying the bladder? (6) A feeling of incomplete bladder emptying? and (7) A feeling of incomplete bowel emptying? The total score is calculated by summing up all responses to the seven-question. The total score ranges from 0 to 28. If the participant’s total response score is greater than zero, she is considered as having symptoms of prolapse.

### Translation and cultural adaptation of POP-SS

The permission to translate the tool was received from the primary author of the original POP-SS [14] (Additional File 1). Translation and cultural adaptation of the POP-SS tool was conducted according to the standard method of translation and adaptation recommendation by Beaton et al. [25]. This standard method includes forward translation, synthesis, backward translation, expert committee review, and pilot testing. The translation is not done on a word-by-word basis. Rather, we considered the geographical context, specific concerns, and cultural meanings that language carries. We gave more emphasis to the back translation because it aids in assessing the translation’s quality [26].

#### Forward and backward translation

Three native *Sidaamu Afoo* speakers (one medical doctor, one reproductive health expert, and one sociologist) who were fluent in English translated the tool from English to *Sidaamu Afoo* independently. A common draft version of *Sidaamu Afoo* was produced from the three translated versions in consensus between the three translators. This version was back-translated into English by two independent translators who were not involved in the forward translation and were fluent in *Sidaamu Afoo* and English. The original and back-translated versions were checked for any discrepancies by the author. Item number 1, “a feeling of something coming down from the vagina” in the original version was stated as ”a feeling of something coming down from the uterus” in the back-translated version. The authors referred back to the translators and identified that the discrepancy happened at the time of forward translation (as they considered using the term “vagina” embarrassing). After correcting this and other- wordings, the first *Sidaamu Afoo* version was produced.

For expert committee review, experts with specialization in gynecology, midwifery, public health, and *Sidaamu Afoo* language reviewed the final forward and backward translations against the original version. For the English word ‘vagina’, a more appropriate word was selected by the committee. Again unnecessarily repeated words were modified (like item number 5, “a difficulty to empty bladder” was stated as “a need to push to completely urinate a urine” on the back-translated version. This was modified as “a need to push to empty bladder”. Then, a pre-final version (version_2) was created. This version was evaluated by six gynecologists from four hospitals for the suitability of each item and relevance for symptom assessment. The agreement was calculated using the Content Validity Index [27].

To evaluate the equivalence and comprehensibility of the translated *Sidaamu Afoo* version, the version_2 tool was also face-validated and pretested among ten women who speak *Sidaamu Afoo* and were admitted to Adare General Hospital and Yirgalem Hamlin Fistula Center with a diagnosis of Pelvic Organ Prolapse (POP). The pretest was conducted by one General Practitioner and one Health Officer working at Yirgalem Hamlin Fistula Center. No change was made after the pretest and version_2 was used as a final version.

### Psychometric evaluation

#### Study participants

The study participants were women aged 18 years and above with symptomatic Pelvic Organ Prolapse who were recruited from the gynecology outpatient department of Yirgalem General Hospital and Yirgalem Hamlin Fistula Center from April to July 2022. Women who were not fluent in *Sidaamu Afoo*, were pregnant (confirmed or suspected), or were early postpartum were excluded.

#### Sample size

To ensure the statistical robustness of the analysis, the sample size was determined based on the recommendation of at least 5–10 subjects per item as per the Consensus-based Standards for the Selection of the Health Measurement Instruments (COSMIN) [28, 29]. Accordingly, the estimated sample size was 70 (10 subjects per item). However, to ensure the adequacy of the sample (to have adequate data for analysis and to obtain precise estimates), we recruited 100 women.

#### Sampling procedure

All women who visited the gynecology outpatient departments of Yirgalem General Hospitals or who were admitted to Yirgalem Hamlin Fistula Center during the study period were assessed for the presence of prolapse symptoms by two important questions: whether they had a feeling of bulging/pressure/something coming down from their vagina or; whether they had a visible mass protruding from the vagina in the past one year [21, 30, 31]. All women who had one or both of the above symptoms were considered symptomatic and invited for pelvic examination to confirm the prolapse. At Yirgalem General Hospital the prolapse symptoms were assessed by a General Practitioner in out-patient department and the pelvic examination was performed by a Gynecologist who is blinded to the questionnaire score. The prolapse stage was classified using the simplified POP-Q system [32, 33]. At Yirgalem Hamline Fistula Center, the symptom assessment was conducted by a Health Officer who took training and was working at Fistula Center. The prolapse stage was classified by a urogynaecologist using the standard POP-Q system [34] which is a very specific and objective system for quantifying and describing POP.

To measure the test-retest reliability, 67 women who can be traced after two weeks (those who have a mobile phone) were purposefully selected based on their accessibility. The same data collector and tool (version_2) was used to collect data about prolapse symptoms through telephone interviews. In this interview, patient stability was not checked. A 2-weeks duration for the retest [28] was preferred because if the duration is too short, they may remember their previous response, which may result in an overestimation of the test-retest reliability. On the other hand, if the time is too long, an individual’s response may be changed due to other interventions and underestimate the test-retest reliability [35].

### Statistical analysis

The collected data were entered into Epi-Data version 3.1 and analyzed using Stata version 16 software [StataCorp LLC, College Station, Texas, USA]. After cleaning and coding the data, sociodemographic characteristics were described by frequency, mean, and standard deviation.

#### Face validity

Face validity, the extent to which the questionnaire is a measure of what it is intended to measure in the opinion of the patient and experts was evaluated by the expert committee through the adaptation process [36]. It is also the degree to which respondents or laypeople believe the questionnaire items are valid. Face validity may drive respondents to answer more accurately, but it is the weakest method of determining a questionnaire’s validity [35, 36]. In this study, the face validity was evaluated first by the experts whether the tool is attractive or not. Then ten women who speak *Sidaamu Afoo* and were admitted to Adare General Hospital and Yirgalem Hamlin Fistula Center with a diagnosis of Pelvic Organ Prolapse (POP) were asked what they thought each question was asking, whether they could paraphrase each question in their terms, and the logic behind their responses [37].

#### Content validity

We evaluated whether the questionnaire could be understood by patients and experts and whether all important and relevant items had been included by the expert panel. Six experts evaluated the comprehensiveness and the relevance using a scale that range from 1 to 4 (1 = not relevant, 2 = somewhat relevant, 3 = quite relevant, and 4 = highly relevant) [38, 39]. Experts’ agreement on relevancy was calculated using the Content Validity Index (CVI), and agreement ≥ 80% was considered acceptable [27, 38]. These experts evaluated each item for content relevance, representativeness, and technical quality.

#### Construct validity

The construct validity was done based on exploratory factor analysis using the Principal Component Analysis (PCA) model. To run the factor analysis, its appropriateness was determined by Kaiser-Meyer-Olkin (KMO) statistics and Bartlett’s test of sphericity. The value of KMO is acceptable if it is > 0.5 [40] and Bartlett’s test is acceptable if it is significant, p-value ≤ 0.05 [41]. To identify the number of meaningful factors, eigenvalues > 1 were considered meaningful and factors were retained for rotation [42]. At the same time, we used a Scree plot indicating eigenvalues for each factor to identify the number of factors to be retained. Then to estimate factor correlations, the varimax orthogonal rotation procedure was applied and commonalities and factor loadings ≥ 0.4 were considered sufficient [43]. Items with factor loading ≥ 0.4 on more than one factor were considered to be cross-loading [40].

#### Convergent validity

To assess how closely a test is related to other tests that measure the same construct (domain), the average inter-item covariance and reliability coefficient were used. Ideally, two tests measuring the same domain (construct) should have high or moderate correlations. A reliability coefficient of (CR > 0.7) and Average variance extracted value (AVE > 0.5) indicates convergent validity [44]. We used average inter-item covariance and correlation of items within each identified domain.

#### Criterion validity

To evaluate the known-group validity, the median difference of POP-SS values among the three stages of POP was compared by using the Kruskal-Wallis test. Significant Kruskal-Wallis’s test indicates the validity of the tool [45]. Furthermore, pairwise multiple comparisons were done by using Dunn’s test to see between which stage of prolapse it can differentiate. A significant level of the test [46] indicates the questionnaires’ ability to differentiate between different stages of the prolapse.

#### Internal consistency and test-retest reliability

The tool’s internal consistency reliability was evaluated using Cronbach’s alpha. Cronbach alpha values between 0.7 and 0.95 [28, 47] indicate adequate internal consistency of the POP-SS questionnaire. Each item’s reliability was also analyzed by assessing its item-total correlation and the overall reliability if a specific item is deleted. Item-total correlation of ≥ 0.5 and inter-item correlation of ≥ 0.3 was considered adequate [48].

Test-retest reliability was computed based on the intra-class correlation coefficient (ICC). To check the test-retest reliability, the data were reshaped from wide form to long form as intra-class correlation coefficient (ICC) estimation works with long data) [49]. Single-rating, absolute agreement, and two-way mixed-effect models were used [16, 49, 50]. An ICC of ≥ 0.7 was considered acceptable [28].

#### Floor and ceiling effects

were computed by percentage frequencies of the lowest and highest scores achieved. Accordingly, ceiling and floor effects were considered present if > 15% of participants achieved this score [28]. The presence of ceiling and floor indicates that the tool fails to discriminate patients at the extremes.

## Results

### Characteristics of study participants

One hundred women volunteered to participate in the study making a response rate of 100%. The age of the participants ranged from 24 to 75 years. Most of the women were from rural areas (91%), can’t read and write (91%), were housewives by occupation (84%), and were Protestant by religion (94%). Fifty-five (55%) had stage 3 prolapse and 81 (81%) of the participants had apical prolapse (Table 1).

**Table 1:** Characteristics of study participants involved in the validation of the *Sidaamu Afoo* version of the Pelvic organ prolapse symptom score tool, Sidama region, Ethiopia, 2022 **(N = 100)**

Variables		Frequency (%) or Mean (±SD)
Age (mean)		46.9 (±10.6)
Residence	Rural	91 (91%)
	Urban	9(9%)
Religion	Protestant	94 (94%)
	Orthodox Christian	4 (4%)
	Muslim	2 (2%)
Marital status	Single	2 (2%)
	Married	64 (64%)
	Widowed	31 (31%)
	Divorced	3 (3%)
Level of education	Unable to read and write	91 (91%)
	Able to read and write	3 (3%)
	Attended school	6 (6%)
Occupation	Housewife	84 (84%)
	Farmers	14 (14%)
	Others	2 (4%)
Number of childbirths (mean)		6 (±2.23)
Women in menopause	Yes	65 (65%)
	No	35 (35%)
Stage of prolapse	Stage 2	37 (37%)
	Stage 3	55 (55%)
	Stage 4	8 (8%)
Prolapse compartment	Apical	81 (81%)
	Anterior	10 (10%)
	Posterior	3 (3%)
	Vault	6 (6%)

### Face validity

Both the panel of experts and POP symptomatic women who participated in the pre-test study confirmed that the tool is easily understandable. No item was suggested to be deleted or added to the scale. The format of the tool remained the same as the original scale and its translated versions (seven questions with five response options).

### Content validity

Six experts evaluated the relevance and comprehensiveness of the questionnaire. The overall content validity index was 0.88 and the Scale Validity index was 0.43. Only One item (item number three) was given an index score of < 0.8. Similarly, a scale validity index of < 0.8 was observed from one expert (expert number 4) (Table 2).

**Table 2:** Content Validity Index score of pelvic organ prolapse symptom score given for the *Sidaamu Afoo* version, June 2022

Item	Expert 1	Expert 2	Expert 3	Expert 4	Expert 5	Expert 6	CVI
A1	1	1	1	1	1	1	1
A2	1	1	1	1	1	1	1
A3	0	1	1	0	1	1	0.68
A4	1	1	1	0	1	1	0.83
A5	1	1	1	1	1	0	0.83
A6	1	1	1	1	1	1	1
A7	1	1	0	1	1	1	0.83
Proportion relevance	0.86	1	0.86	0.71	1	0.86	Mean CVI = 0.88

*CVI = Content Validity Index *SVI (Scale Validity Index) = 0.43

### Reliability and item analysis

The overall internal consistency of the POP-SS questionnaire was 0.79 (95% confidence interval of 0.73–0.85). There was no evidence of an increase in this value if an item is deleted. The percentage distribution of the responses showed that an answer of zero (0) was given most frequently to one item (A feeling of incomplete bowel emptying) (51%). An answer of four [4] was given most frequently to two items (A feeling of something coming down from your vagina [71%] and A feeling of discomfort /pain in the vagina [72%]). Item-total correlation, inter-item correlation coefficient; and floor and ceiling effects are presented in Table 3. Among the 67 women who were chosen for a second-round interview two weeks after the first interview, six of them were not accessible. Hence, only 61 women participated in the re-test interview. The test-retest reliability value was 0.83 (95% CI: 0.50–0.92) (Table 3).

**Table 3:** Internal consistency, test-retest reliability; and ceiling and floor effect score of the *Sidaamu Afoo* version of pelvic organ prolapse symptom score, June 2022

Items	Item-total correlation	Inter-item correlation	Internal consistency (95% CI) (A1-A7)	Ceiling (n)	Floor (n)	Test-retest values		
						(ICC3,1)	95%CI	P _value
A1. A feeling of something coming down from the vagina	0.27	0.78	**0.79 (0.73, 0.85)**	**71**	0	0.83	(0.50, 0.92)	< 0.001
A2. An uncomfortable feeling or pain in your vagina.	0.31	0.78		**72**	2	0.66	(0.48, 0.79)	< 0.001
A3. A heaviness or dragging feeling in your lower abdomen.	0.61	0.72		7	21	0.78	(0.63, 0.87)	< 0.001
A4. A heaviness or dragging feeling in your lower abdomen.	0.67	0.70		12	16	0.78	(0.59, 0.88)	< 0.001
A5. A need to strain (push) to empty your bladder	0.55	0.73		18	25	0.81	(0.66, 0.89)	< 0.001
A6. A feeling that your bladder has not emptied completely	0.54	0.73		17	22	0.75	(0.61, 0.84)	< 0.001
A7. A feeling that your bowel has not emptied completely	0.51	0.74		6	**51**	0.81	(0.65, 0.90)	< 0.001

*(ICC3, 1) = Single rating, Two-way Mixed-effects model (absolute agreement); CI = confidence interval

### Construct validity

**Fig. 1:**
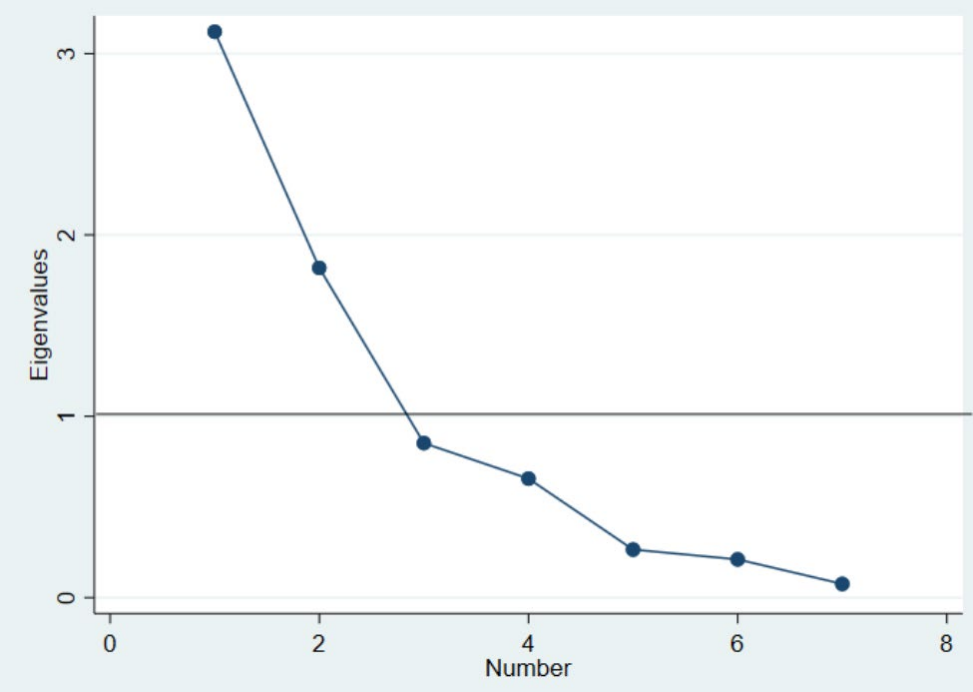
A scree plot indicating the number of factors to be retained for the Sidaamu Afoo version of the POP-SS questionnaire, June 2022

Bartlett’s test of sphericity (χ^2^ = 412.53, p < 0.001) and the Kaiser-Meyer-Olkin measure of sampling adequacy showed that the POP-SS has adequate common variance (KMO = 0.66) for factor analysis. Using the principal component analysis model, the factor analysis extracted two factors (components) based on a minimum eigenvalue of (1) The factors were rotated orthogonally by using a varimax approach to easily interpret factor loadings and identify items that loaded on each factor (component). Two items (*feeling of something coming down from the vagina* and *an uncomfortable feeling or pain in your vagina*) loaded to Factor 2 with factor loadings of 0.87 and 0.92, respectively. Five items (*a heaviness feeling in the lower abdomen, a dragging feeling in the lower back, difficulty in emptying the bladder, a feeling of incomplete bladder emptying and a feeling of incomplete bowel emptying*) loaded to Factor 1 with factor loadings ranging from 0.61 to 0.90 (Table 4). Factor 1 explained 44.6% of the common variance, and Factor 2 explained 26%. Both factors combined explained 70.6% of the common variance. The eigenvalue was 3.1 for Factor 1 and 1.8 for Factor (2) The scree plot constructed based on eigenvalues of 1 also indicated two factors (dimensions) of the *Sidaamu Afoo version* of the POP-SS (Fig. 1). Communalities for each item ranged from 0.44 to 0.84, indicating adequate commonalities.

**Table 4:** Construct and convergent validities of the *Sidaamu Afoo* version of pelvic organ prolapse symptom score, June 2022

POP-SS Items	Construct validity			Convergent validity		
	Item-loading	Variance (%)	Eigenvalue	**Reliability coefficient**	**Average inter-item covariance**	**Scale reliability coefficient**
**Factor 1**						
A1. A feeling of something coming down from the vagina	0.02	**44.6**	**3.1**		**0.89**	**0.817**
A2. An uncomfortable feeling or pain in your vagina.	0.01					
A3. A heaviness or dragging feeling in your lower abdomen.	**0.74**			0.81		
A4. A heaviness or dragging feeling in your lower abdomen.	**0.74**			0.80		
A5. A need to strain (push) to empty your bladder	**0.90**			0.78		
A6. A feeling that your bladder has not emptied completely	**0.86**			0.80		
A7. A feeling that your bowel has not emptied completely	**0.61**			0.85		
**Factor 2**						
A1. A feeling of something coming down from the vagina	**0.87**	26	1.8	0.71	0.37	**0.817**
A2. An uncomfortable feeling or pain in your vagina.	**0.92**			0.71		
A3. A heaviness or dragging feeling in your lower abdomen.	0.29					
A4. A heaviness or dragging feeling in your lower abdomen.	0.36					
A5. A need to strain (push) to empty your bladder	0.17					
A6. A feeling that your bladder has not emptied completely	0.13					
A7. A feeling that your bowel has not emptied completely	0.27					
Total		**70.6**				

### Convergent validity

The correlation of items within each factor (convergent validity) assessed by the internal consistency reliability of each factor has an adequate correlation (scale reliability coefficient) of 0.84 for Factor 1 and 0.82 for Factor 2. The average inter-item covariance was 0.89 in Factor 1 and 0.37 in Factor 2 (Table 4).

### Criterion validity: known groups validity

The criterion validity showed that there was a significant difference in the median of POP-SS among the three groups of prolapse stages based on the Kruskal-Wallis test (Kruskal-Wallis χ^2^, 17.5, p < 0.001). In addition, pairwise multiple comparisons showed that the tool can differentiate stage 2 prolapse from stage 3 (p < 0.001) and stage 2 from stage 4 (p = 0.011), but no significant difference was observed between stage 3 and stage 4 (p = 0.46). The median of POP-SS was 12 in stage 2 patients, 18 in stage 3 and 19 in stage 4 participants with POP.

## Discussion

The Pelvic Organ Prolapse Symptom Score (POP-SS) tool was successfully translated into *Sidaamu Afoo* language and achieved adequate psychometric properties. The tool has a content validity index of 0.88, internal consistency reliability (Cronbach alpha) of 0.79, and test-retest reliability (ICC) of 0.83. Bartlett’s test of sphericity was significant and the Kaiser-Meyer-Olkin (KMO) statistics was 0.66 indicating the adequacy of the sample for factor analysis. Factor analysis revealed two factors with a common variance of 70.6% and factor loadings ranged from 0.61 to 0.92. Evaluation of criterion (known-group) validity showed that the current version of POP-SS can differentiate between POP stages. It was able to differentiate between stages 2 and 3, and between stages 2 and 4, but not between stages 3 and 4.

During the translation of this tool, the changes were made more at the level of the meaning of the sentences considering culture rather than more specific word modifications to maintain the original meaning and context. More attention was given to the back-translation as it helps in assessing the translation’s quality [26]. These efforts helped to maintain the meaning and purpose of the tool similar to the original version which in turn will help for international comparisons. The evaluation made by a panel of experts and POP symptomatic women suggests maintaining the questionnaire format as it is (i.e., no item was suggested to be deleted or added to the scale). This indicates the current, *Sidaamu Afoo*, version is technically equivalent to the original scale and its translated versions [14, 20–22] in terms of the number of items [7], response options (0 to 4), and outcome measure (a symptom of prolapse). The *Sidaamu Afoo* version had an adequate content validity index (0.88) and Scale Validity Index (0.43). This shows the agreement among experts on the relevance of the items and the comprehensiveness of the overall scale.

The questionnaire also had acceptable internal consistency reliability (Cronbach alpha = 0.79). This value is in-line with the values of the original tool (0.72–0.83) based on the source of data used [14] and the Chinese version (0.78) [22], but slightly lower than the result of the Amharic version (0.86) [21] and higher than the result of the Turkish version (0.71) [20]. Despite the slight difference in the magnitude of the reliability coefficient, all values are greater than the threshold value (0.7) [47], indicating that the tool is internally consistent. The test-retest reliability of the questionnaire was also good (ICC = 0.83). This value is similar to the result of the Amharic version (0.81) [21] but lower in size compared to the Chinese and Turkish versions (0.98) [20, 22]. Still, the result of these findings indicates that the scale is consistent over time.

The exploratory factor analysis identified two factors (components) based on a minimum eigenvalue of one. The first factor contains items number three to seven and the second factor contains the first two items: *a feeling of something coming down from the vagina* and *discomfort feeling/pain in the vagina*). The first two items (symptoms) are a direct result of something protruding from the vagina. So, this construct (dimension) can be named protrusion-related symptoms. The remaining five items (symptoms) can occur when the prolapsing part is within the vaginal canal (without protruding out of the vagina) and can be named non-protrusion symptoms.

The number of factors identified is similar to all the previous three versions [20–22]. The original version did not report on factor analysis, but the translated three versions named the two factors as physical and evacuation symptoms. However, the last item (a feeling of not completely emptying the bowel) inconsistently loaded to each factor. In this study, only two items (*a feeling of something coming down from the vagina* and *discomfort feeling/pain in the vagina*) loaded to one factor and the remaining five items loaded to the other factor. This might be because the majority (63%) of the current study participants were women who were diagnosed with advanced-stage of prolapse. In an advanced stage of prolapse, protrusion of mass per vagina and discomfort in the vagina exist always [20, 51–53], but the other symptoms may not exist always. The items in each factor had good factor loadings, indicating a good correlation with the underlying factor. Yong and Pearce suggest that a factor with two variables (items) can be considered reliable if the variables (items) are highly correlated (r > 0.7) but fairly uncorrelated with other items [54]. This tool has a convergent validity in terms of Cronbach alpha of 0.84 in Factor 1 and 0.82 in Factor 2. This indicates the items in each factor (construct) are highly correlated. The two factors explained 70.6% of the common variance. This is similar to the Amharic version (71.56%) [21] and higher than the Turkish version 55.9% [20].

Based on the Kruskal-Wallis test, the criterion validity test shows that the *Sidaamu Afoo* version of POP-SS can produce significantly different POP symptom scores across the different stages of prolapse confirmed by urogynaecologist. Specifically, it was able to differentiate between the early prolapse stage (stage 2) and advanced stages (stages 3, p < 0.001; and stage 4, p = 0.011). However, no significant difference was observed between the advanced stages (stages 3 and 4, p = 0.46). This could be because stages 3 and 4 are sometimes interchangeable depending on the presence of factors aggravating the prolapse like heavy work at the time of diagnosis. Since the management protocol is similar for advanced stages (stages 3 and 4), this tool has clinical importance in diagnosing the patient for POP and monitoring prolapse symptoms. The Turkish and Amharic versions also showed that the highest stage of prolapse had a higher value of POP-SS [20, 21]. Both long-term and short-term follow-up is necessary even after surgery for pelvic floor disorders to evaluate treatment success [12, 55]. Therefore, this tool has importance in diagnosing POP, for patient follow up after treatment and also to compare effectiveness of different treatment options.

The ceiling and floor effect test showed that an answer of zero (0) was given most frequently to one item (*a feeling of incomplete bowel emptying*, 51%). This agrees with the result of the Amharic version of the POP-SS (73) [21]. This score (zero) might be due to the fewer number (3%) of participants with posterior prolapse in this study. Unless the posterior compartment is involved in the prolapse, a feeling of incomplete bowel emptying may not occur. On the other hand, a maximum rating of four [4] was given most frequently to two items (*a feeling of something coming down from your vagina* [71%] and *a feeling of discomfort /pain in the vagina* [72%]). The result of the Turkish version of POP-SS [20] also showed that the ceiling effect was seen in a similar item. In the current study, there is no lower score rating given for item number one (feeling of something coming down from your vagina). This might be again due to the majority of the participants having advanced stages of prolapse.

To our knowledge, this is the first validation of the *Sidaamu Afoo* version of the POP-SS tool. However, it is not free of limitations. This study did not involve women with prolapse stage one because we did not get them during the study period. However, since stage 1 is not considered clinically relevant and most women seek healthcare only when the prolapse is advanced [56], this tool is valid and can be used in clinical settings. The other limitation is that the criterion validity of the tool was evaluated only against the known group validity test, but not compared against other standard tools like the Prolapse-Quality of life tool and Pelvic Floor Distress Inventory (PFDI-20) tool. Thirdly, the lack of participants in stage one and the inclusion of a majority from the advanced stage of prolapse resulted in a considerable number of floor and ceiling effects. It has also produced only two items that loaded to Factor 2 with lower average inter-item covariance. Further, most (91%) of the study participants can’t read and write, so the questionnaire was administered through interviews. The way the questions were communicated during interviews might influence participants’ responses in a way that may produce bias.

## Conclusion

The *Sidaamu Afoo* version of POP-SS was successfully translated, and it is consistent, valid, and reliable to use for the assessment of pelvic organ prolapse both in research and clinical setups. Further, studies that involve a balanced number of women in each stage of prolapse are needed to evaluate the ceiling and floor effects.

## Electronic supplementary material

Below is the link to the electronic supplementary material.

Additional File 1: Pelvic Organ Prolapse Symptom Score

## Data Availability

All data on which the results are based are provided within the manuscript.

## Abbreviations

ICC: Intra-class Correlation Coefficient
POP: Pelvic Organ Prolapse
POP-SS: Pelvic Organ Prolapse Symptom Score
