# Supplementary material for: Reliability and validity of the Sidaamu Afoo version of the pelvic organ prolapse symptom score questionnaire
Source: BMC Womens Health. 2023 Jun 20;23:324. doi: 10.1186/s12905-023-02478-x (PMC10283297; doi:10.1186/s12905-023-02478-x)
Supplement: Supplementary file 1 — Additional File 1: Pelvic Organ Prolapse Symptom Score [file 12905_2023_2478_MOESM1_ESM.pdf]

Pelvic Organ Prolapse Symptom Score

This symptom score can be used from time to time to help you check how your prolapse symptoms are changing.

Answer the following questions, thinking about how you have been, on average, over the **PAST FOUR WEEKS**. ***(Please cross one box in each row)***

**How often during the last four weeks have you had the following symptoms:**		**Never**	**Occasion-ally**	**Some-times**	**Most of the time**	**All of the time**
**Q1**	a feeling of something coming down from or in your vagina?	☐ 0	☐ 1	☐ 2	☐ 3	☐ 4
**Q2**	an uncomfortable feeling or pain in your vagina which is worse when standing?	☐ 0	☐ 1	☐ 2	☐ 3	☐ 4
**Q3**	a heaviness or dragging feeling in your lower abdomen (tummy)?	☐ 0	☐ 1	☐ 2	☐ 3	☐ 4
**Q4**	a heaviness or dragging feeling in your lower back?	☐ 0	☐ 1	☐ 2	☐ 3	☐ 4
**Q5**	a need to strain (push) to empty your bladder?	☐ 0	☐ 1	☐ 2	☐ 3	☐ 4
**Q6**	a feeling that your bladder has not emptied completely?	☐ 0	☐ 1	☐ 2	☐ 3	☐ 4
**Q7**	a feeling that your bowel has not emptied completely?	☐ 0	☐ 1	☐ 2	☐ 3	☐ 4

Q8 which of the symptoms above (questions Q1 to Q7) causes you most bother? **Q** ☐ Not applicable ☐
Please enter a number from 1 to 7 in the box, or cross "Not applicable"

Copyright © 2008 S Hagen
